# Cardiovascular and Quality of Life Outcomes of a 3-Month Physical Exercise Program in Two Brazilian Communities

**DOI:** 10.3389/fmed.2020.568796

**Published:** 2020-10-20

**Authors:** Marcelo Pereira de Lima, Severo Conopca, Renata Miyabara, Geovanna Romeiro, Luciana A. Campos, Ovidiu C. Baltatu

**Affiliations:** ^1^Center of Innovation, Technology and Education (CITE), Sao Jose dos Campos Technology Park, Sao Jose dos Campos, Brazil; ^2^Institute of Biomedical Engineering, Anhembi Morumbi University - Laureate International Universities, Sao Jose dos Campos, Brazil; ^3^Physiotherapy School, University Centre of Espirito Santo, Colatina, Brazil; ^4^Department of Health, Santa Rita University Center, São Paulo, Brazil; ^5^College of Health Sciences, Abu Dhabi University, Abu Dhabi, United Arab Emirates; ^6^College of Medicine & Health Sciences, Khalifa University, Abu Dhabi, United Arab Emirates

**Keywords:** quality of life, physical exercise (EX), cardiovascular risk (CV risk), aging–old age–seniors, community activities

## Abstract

**Background:** A reduction in physical activity levels in older people is associated with declining quality of life and lower cardiorespiratory fitness levels associated with cardiovascular disease outcomes and mortality from all causes. Evidence supports the positive effect of community-based exercise (CEXE) programs on cardiovascular health and quality of life. This research aimed to examine the effects of a 3-month CEXE on health-related quality of life and cardiovascular risk factors in two Brazilian populations.

**Methods:** Adults with an average age of 70.2 ± 5.4 years were recruited to engage in an individually designed group based CEXE program two to three times/week (aerobic exercise, circuit resistance training, and stretching exercises for 1 h each time). Once a week, competitions were held to improve socialization and collaboration capacity among group members. The CEXE group was compared with a sedentary group. Cardiovascular outcomes were blood pressure, triglycerides, body mass index, waist circumference, high-density lipoprotein cholesterol (HDL-C), low-density lipoprotein cholesterol, total cholesterol, and glycemia. Health-related quality of life was evaluated using the Short Form-36.

**Results:** Of the cardiovascular outcomes studied, the CEXE program significantly reduced systolic blood pressure [5.7 (95% CI 0.2 to 11.3), *p* < 0.05] and the triglyceride–HDL-C ratio [0.8 (95% CI 0.05 to 1.5), *p* < 0.05], whereas HDL-C was significantly increased [4.4 (95% CI 0.02 to 8.8), *p* < 0.05]. A significant improvement in the Short Form-36 subscales occurred in CEXE but not in the control group: physical functioning score [increase of 24.2 (95% CI 11.8 to 36.5) vs. −9.2 (95% CI −21.5 to 3.2), *p* < 0.001], physical role functioning score [increase of 35.4 (95% CI 12.8 to 58.0) vs. 16.7 (95% CI −6.0 to 39.3), *p* < 0.01], and general health score [increase of 23.7 (95% CI: 36.9. to 10.4) vs. 2.4 (95% CI −10.9 to 15.7), *p* < 0.001].

**Conclusion:** This study shows that in older adults, a 12-week physical activity program can significantly decrease cardiovascular risk and improve health-related quality of life measures. An important transferable sociocultural strategy of our exercise program was to establish social interactions during and outside the CEXE program.

## Background

Increases in both longevity and older adults have been observed globally over the past years. The rise in life expectancy increases public health demands due to a higher number of physically impaired older adults suffering a decline in quality of life (QoL) and an increasing burden of cardiovascular diseases (1). Physical inactivity is among the top 10 risk factors for global disease burden reaching a pandemic status (2, 3). This condition aggravates with age, from 45% of physically inactive people aged 60 years to 75% at age 75 years (4). A decline in functional fitness in older women is related to worsening of QoL (5) and a lower cardiorespiratory fitness level, which are associated with cardiovascular disease events and mortality from all causes (6). Besides Brazil and Latin America, the prevalence of cardiovascular diseases associated with physical inactivity of older adults is growing worldwide, particularly in women (7, 8).

Growing evidence from high-quality research supports improved health in older adults through physical activity (9). The American College of Sports Medicine's position summarizes the advantages of both long-term exercise and physical activity and shorter-duration exercise programs (EXEs) on health and functional capacity for older adults (10). Developing and implementing physical activity programs in older adults, however, is a real challenge for health professionals. Adherence to physical activity guidelines for older adults is poor, and few meet existing physical activity guidelines (11).

According to the American College of Sports Medicine's Position Stand, the impact of exercise on physical performance belongs to category C/D, meaning it is overlooked and does not seem linear, and more research is required to understand the precise nature of the relationship between exercise and functional performance. Moreover, the effects of physical activity on QoL in older adults belong to category D research, indicating that although physical activity seems to be positively correlated with certain aspects of QOL, the precise nature of their relationship is poorly understood (10).

This study aimed to investigate the impact of a community-based short-term (12-week) physical EXE on cardiovascular health and QoL in older adults.

## Methods

### Study Setup and Outcome Measures

The target population for the community-based physical EXE was community-dwelling older people with an insufficiently active lifestyle. This was a prospective, longitudinal, and parallel-assigned study. All medical examinations were conducted with patient consent. Anhembi Morumbi University and Brasil University's Institutional Ethical Committee approved this study (CAAE: 11818919.8.0000.5492, and 24558913.7.0000.5494, respectively). Follow-up data were obtained by retrospective anonymized chart review and analyzed.

The primary outcome measures were health-related quality of life (HRQoL), and the secondary outcomes were changes in cardiovascular risk factors. The timeline for outcome assessments was at baseline and after 12 weeks of the exercise intervention program. Outcome assessors were masked to the group allocation.

The following cardiovascular risk measures were investigated: arterial blood pressure, body mass index (BMI), waist circumference, triglycerides, high-density lipoprotein cholesterol (HDL-C), and fasting glycemia. Blood pressure was measured in the non-dominant arm with the individual seated and resting for at least 5 min. Bodyweight was obtained on an electronic scale with the individual wearing only light clothing and with an empty bladder. Height was obtained using a wall stadiometer with the individual barefooted, and the BMI (BMI = weight/height^2^) was calculated. Waist circumference was measured at a level midway between the lowest rib and the iliac crest. Biochemical examinations were performed in a clinical laboratory.

QoL was evaluated using Short Form-36 validated in Brazil and adapted to socioeconomic and cultural conditions (12). The Short Form-36 consists of 36 questions yielding an eight-scale profile of functional health and well-being scores: general health perceptions, physical functioning, physical role functioning, vitality, social functioning, emotional role functioning, mental health, and bodily pain.

### Participants in the Study—Inclusion and Exclusion Criteria

Older adults aged more than 60 years from two municipalities participated in the study: one group was in the municipality of Ourinhos, São Paulo, and registered with UNIMED; the second group was at Guaratinga, Bahia, and registered at the Municipal Department of Health. Inclusion criteria were women and men aged over 60 years, willing and able to engage in moderate forms of physical activity, voluntary participation, insufficiently active, defined as a person who does not meet the physical activity recommendations of the World Health Organization: 30 min per day of moderate physical activity (including active leisure and travel), 5 days a week; or 30 min per day of vigorous physical activity, 3 days a week. Exclusion criteria were a serious disease that limits physical activity (cardiovascular or respiratory disease, major neuromuscular disease, cancer, or recent major surgery), psychological disease, and prescription of neuroleptic medication.

### Physical Exercise Intervention Program

The community-based physical EXE adhered to the American College of Sports Medicine guideline, as it involves combined aerobic exercise, muscle strengthening exercises, and flexibility exercises (10). A progressive and structured exercise training was 50–60 min per session at an intensity of 1 to 3 in the rate of perceived exertion, two to three times a week, for 12 weeks. The sessions involved a combination of aerobic, strength, balance, and flexibility exercises guided by a physical therapist and physical education instructor. To ensure the commitment of participants to our physical activity program, we aimed at “(1) increasing awareness of benefits and reducing perceived risks of physical activity and (2) enhancing environmental and financial access to physical activity opportunities,” as suggested by Franco et al. (4). An important positive feature of our physical EXE was participants' opportunity to socially interact and enjoy group exercise, as McPhate et al. (13) defined.

The training session started with a general warm-up (articular and cardiorespiratory), 10-min mats, and flexibility exercises. The second step consisted of 20-min aerobic exercise through a group walk around the gymnasium's sports court, with intensity from mild to moderate, respecting each participant's limit. The next step was to strengthen the lower and upper extremities for 20 min of alternating exercises and use canes, dumbbells, ankle weights, and elastic bands. Closing the exercise was focused on stretching the major muscle groups for 10 min, keeping two replications for 30 s per muscle group. Once a week, competitions were held to improve socialization and collaboration capacity among group members. These competitions were in the form of a circuit of static and dynamic exercises with similar movements required to perform basic everyday tasks, games perception, and body awareness. Control group participants offered regular advice on healthy behaviors.

### Statistical Analysis

The D'Agostino & Pearson omnibus and Kolmogorov–Smirnov tests (with Dallal–Wilkinson–Lillie for corrected *P*-value) were used to assess data normality. Mixed-effect analysis followed by Fisher's multiple comparison test to study the importance of the physical exercise intervention program in the active group relative to the control group (GraphPad Prism version 8.2.1 for Mac OS X, GraphPad Software, La Jolla California USA, www.graphpad.com). Data are presented as the mean and standard error of the mean. Differences were considered significant when the probability of a type I error was lower than 5% (*p* < 0.05).

## Results

Eighty-five (85) study participants completed the study: 42 EXE participants and 43 control (sedentary) volunteers who did not participate in EXE. The recruited cohort included women (60%, 51 women) and men aged 69.9 ± 0.9 years (EXE group) and 70.2 ± 0.8 years (control group).

**Figure 1:**
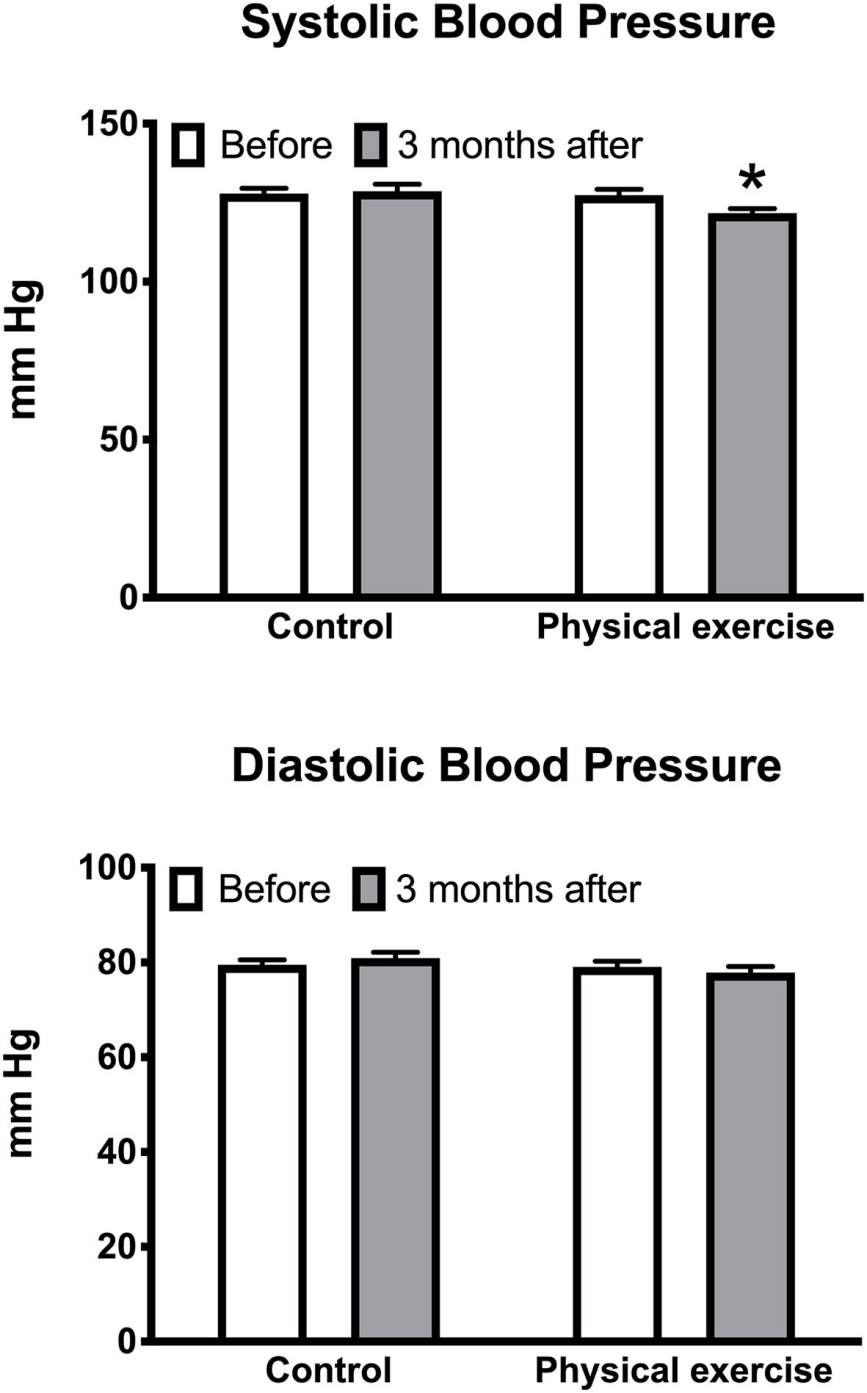
Effects of the physical exercise program on systolic and diastolic blood pressure. Data are mean with standard error; *p < 0.05.

**Figure 2:**
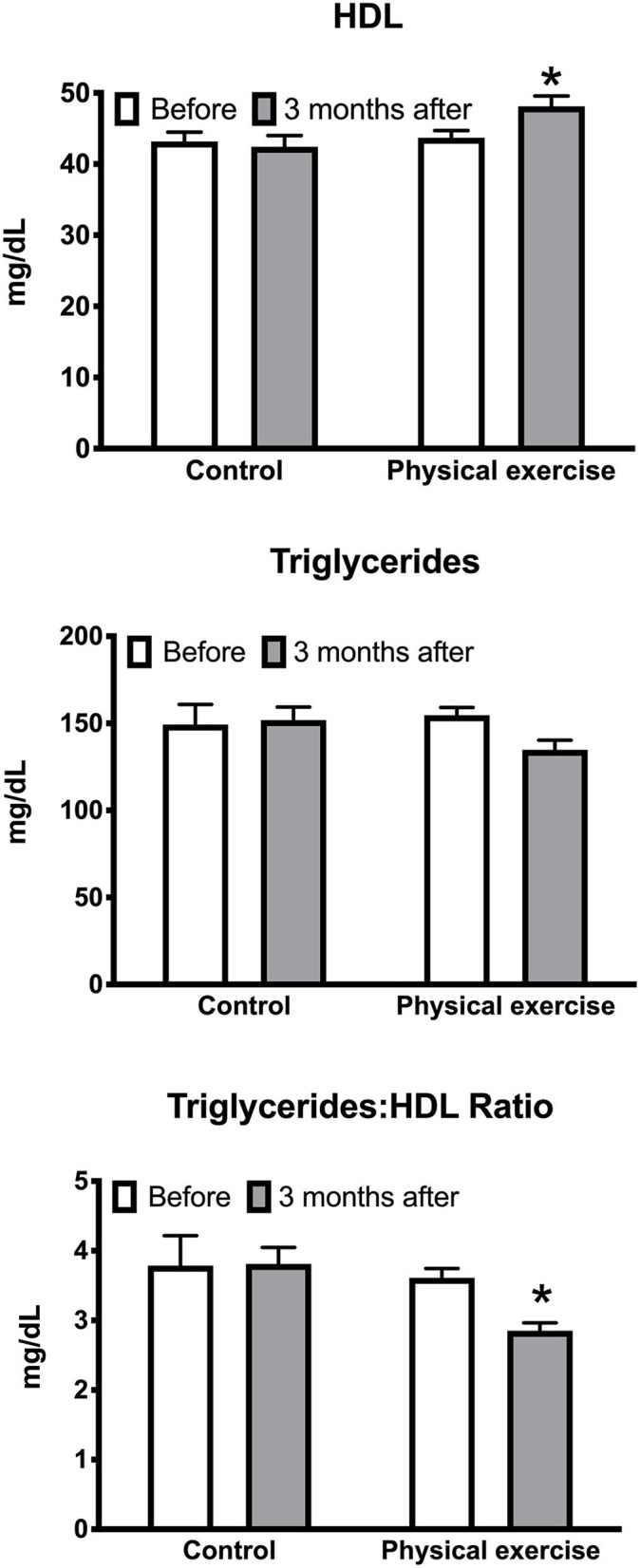
Effects of the physical exercise program on HDL-C, triglycerides, and triglyceride:HDL-C ratio. Data are mean with standard error; *p < 0.05.

Of the investigated cardiovascular outcome measures, significantly decreased by the EXE program were systolic BP [5.7 (95% CI 0.2 to 11.3), *p* < 0.05] (Figure 1) and the triglyceride–HDL-C ratio [0.8 (95% CI 0.05 to 1.5), *p* < 0.05], whereas HDL-C was significantly increased [4.4 (95% CI 0.02 to 8.8), *p* < 0.05] (Figure 2). No significant effects of the EXE were observed for diastolic BP, BMI, waist circumference, total cholesterol, LDL-C, triglycerides, or fasting glucose (Table 1).

**Table 1:** Cardiovascular risk and health-related quality of life measures.

**Outcome measures**	**Sedentary group**		**Physical exercise group**	
	**Before**	**After**	**Before**	**After**
Age (years)	70.2 ± 0.8		69.9 ± 0.9	
BMI (kg/m^2^)	29.7 ± 0.8	29.7 ± 0.7	28.1 ± 0.6	28.3 ± 0.6
Waist circumference (cm)	100.7 ± 1.9	100.5 ± 1.8	93.5 ± 1.7	93.1 ± 1.6
Blood pressure systolic^*^/diastolic (mm Hg)	127.9 ± 1.6/79.5 ± 1.2	128.6 ± 2.2/80.9 ± 1.2	127.4 ± 1.8/79.0 ± 1.2	121.6 ± 1.4/77.9 ± 1.3
Total cholesterol (mg/dl)	196.1 ± 7.9	196.1 ± 7.9	191.6 ± 6.8	193.8 ± 6.7
Triglycerides (mg/dl)^*^	149.3 ± 11.5	151.8 ± 7.5	154.7 ± 4.4	134.8 ± 5.5
HDL-C (mg/dl)^*^	43.2 ± 1.3	42.4 ± 1.6	43.7 ± 1.0	48.1 ± 1.4
LDL-C (mg/dl)	111.7 ± 6.8	123.4 ± 7.6	122.9 ± 7.6	123.3 ± 6.6
Triglyceride:HDL-C ratio^*^	3.8 ± 0.4	3.8 ± 0.2	3.6 ± 0.1	2.8 ± 0.1
Fasting glucose	101.7 ± 3.4	114.3 ± 7.4	100.1 ± 3.4	105.3 ± 3.2
**Health-related quality of life (HRQoL) scores**				
General health	62.6 ± 5.7	65.0 ± 6.5	49.4 ± 6.5	73.1 ± 3.1
Vitality	52.9 ± 6.6	56.7 ± 9.2	59.6 ± 5.9	64.6 ± 4.5
Physical function	52.9 ± 9.4	43.7 ± 8.0	50.8 ± 7.0	75.0 ± 4.7
Role physical	22.9 ± 11.3	39.6 ± 10.9	37.5 ± 11.7	72.9 ± 8.4
Social function	65.6 ± 7.2	64.6 ± 9.7	83.3 ± 6.7	81.7 ± 4.5
Emotional role	52.8 ± 14.5	44.4 ± 12.5	55.6 ± 13.2	75.0 ± 8.3
Mental health	63.3 ± 4.1	66.0 ± 8.2	70.7 ± 6.9	81.7 ± 4.0
Bodily pain	44.6 ± 7.7	55.7 ± 6.6	61.9 ± 7.5	70.6 ± 3.9

* *Significant differences in outcome measures by mixed-effects analysis are presented in figures*.

**Figure 3:**
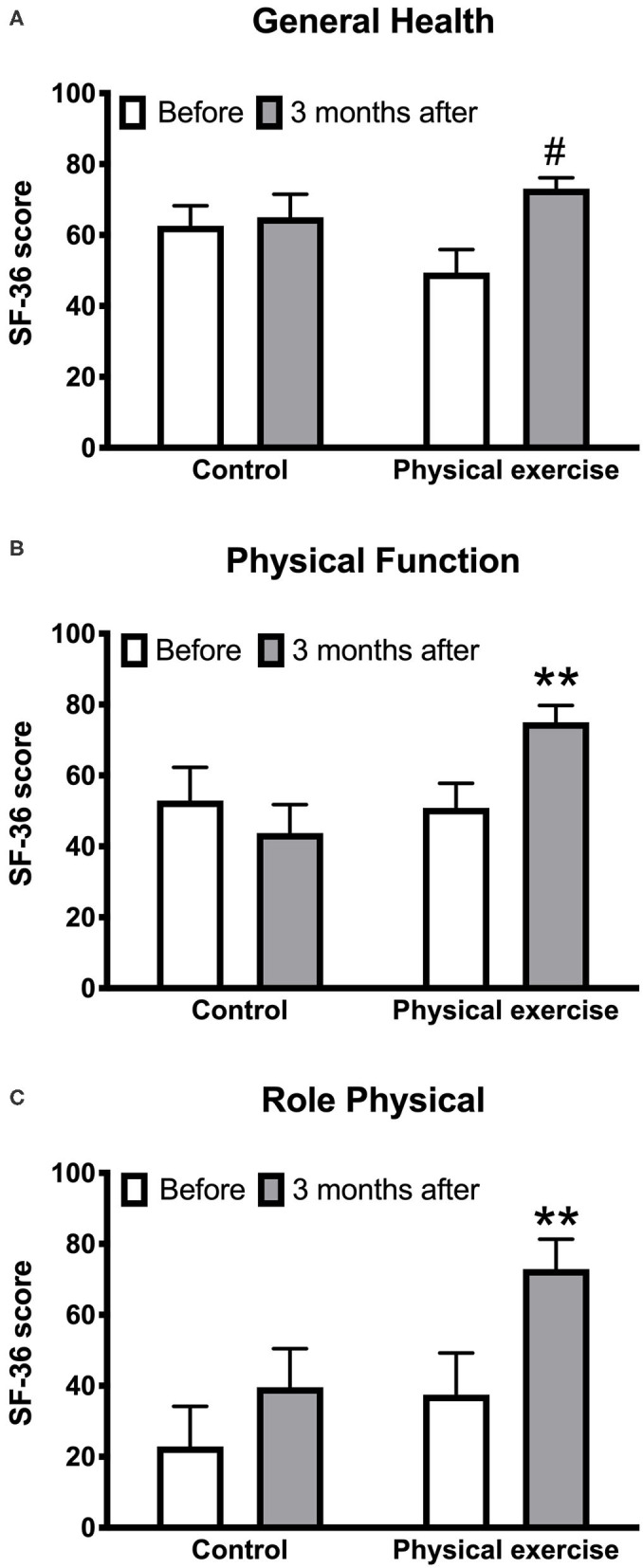
Effects of the physical exercise program on the health-related quality of life measures: general health score **(A)**, physical function score **(B)**, physical role score **(C)**. Data are mean with standard error; **p < 0.01; ^#^*p* < 0.001.

Significant improvements were observed in the following HRQoL measures in the exercise but not in the control group:

° physical functioning score [increase of 24.2 (95% CI 11.8 to 36.5) vs. −9.2 (95% CI −21.5 to 3.2), *p* < 0.001] (Figure 3A),
° physical role functioning score [increase of 35.4 (95% CI 12.8 to 58.0) vs. 16.7 (95% CI −6.0 to 39.3), *p* < 0.01] (Figure 3B), and
° general health score [increase of 23.7 (95% CI 36.9 to 10.4) vs. 2.4 (95% CI −10.9 to 15.7), *p* < 0.001] (Figure 3C).

There were no differences between study groups and intervention in the following HRQoL measures: vitality, social function, and emotional role (Table 1).

## Discussion

The main outcome of this study is that a 12-week community-based physical EXE may significantly improve certain cardiovascular risk factors and HRQoL measures, including systolic BP, HLD-C, triglyceride–HDL-C ratio, physical capacity, physical function, and general health scores.

Combined training protocols with aerobic and resistance exercise are recommended as non-pharmacological therapies to prevent hypertension and seem to be a better fit for the aged, deconditioned, and hypertensive individuals (14). Physical exercise can significantly improve cardiorespiratory fitness and some cardiometabolic biomarkers in adults without cardiovascular disease. Moreover, exercise has a beneficial impact on many of the known cardiovascular disease risk factors, such as hypertension, dyslipidemia, and type II diabetes (15). Physical exercise, for example, reduces blood pressure, LDL-C, and triglycerides, enhances glucose–insulin homeostasis, and raises HDL-C (16). In our study, systolic BP significantly decreased. Higher triglyceride:HDL-C ratio and lower HDL-C correlate with cardiovascular disease risk (17). Several studies showed that physical exercise could play an important role in preserving HDL-C concentrations in older people (18). A 1% decrease in HDL-C was associated with a 2–3% rise in cardiovascular risk (19).

The effects of exercise training on the lipoprotein profile and hemodynamic parameters were demonstrated. Studies involving low and moderate exercise training in apparently healthy adults over 8 weeks resulted in a significant reduction in total cholesterol (20). Exercise training in older people with heart failure has been recognized as a key component of the cardiac rehabilitation program (21). Exercise-based cardiac rehabilitation programs improve functional and hemodynamic parameters and induce an antioxidant response (22, 23).

Based on our results, it is reasonable to conclude that involvement in a physical activity program of at least 12 weeks will be sufficient to decrease the risk of cardiovascular disease, as indicated by decreased systolic BP, triglyceride:HDL-C ratio, and rise in HDL-C. Because the effects on triglyceride:HDL-C ratio and HDL-C occurred without concurrent weight or diet adjustments, this may suggest that the EXE alone will alter the lipoprotein profile in older people. The other cardiovascular risk factors studied, including triglycerides, BMI, and waist circumference, were not affected by the EXE, indicating the need for a training duration of more than 12 weeks to detect changes in these measures. This is consistent with category A evidence that three or more months of moderate-intensity exercise result in cardiovascular changes in stable older adults (10). Also, preventive measures, including eating patterns and lifestyle changes, should be considered.

Sedentary behaviors are independently associated with physical, functional, mental, and cognitive health among older adults in retirement communities (24). Our physical exercise training program increased physical capacity, physical function, and general health. Our research also promotes the impact of a combined physical EXE on HRQoL measures in older people. Specifically, our program significantly improved physical and general health domains. These physical domains seem to be strong and independent predictors of long-term cardiovascular events (25). Furthermore, HRQoL metrics are correlated with the cardiovascular risk factors studied, including BMI and waist circumference (26). HRQoL indicators can be considered cardiovascular risk factors in addition to the Framingham five main modifiable risk factors: smoking, hypertension, diabetes, high cholesterol and obesity, and two non-modifiable risk factors—age and sex (27).

An important component for implementing our physical exercise intervention program effectively was recognizing the factors affecting the physical activity behavior of participants. We agree with the identified six factors that influence the behavior of physical activity identified by Franco et al. (4): social influences, physical limitations, competing priorities, access difficulties, personal benefits of physical activity, and motivation and beliefs. The Group-Based Physical Activity for the Older Adult Trial of Beauchamp et al. (28) suggests that community EXEs should attempt to engage in age-targeting but not necessarily sex-targeting among older adults. Our physical exercise community program aimed at resolving these factors.

Exercise training programs that consider social, environmental, and cultural factors to increase physical activity are needed to contrast sedentary habits (29). Greater efforts are needed to prevent sedentary behavior and physical inactivity while promoting physical activity in all age categories (30). For older people, a multicomponent EXE is considered the most suitable for both free-living and community settings (31).

In summary, we conclude that a 3-month physical exercise intervention program was successful in reducing cardiovascular risk and enhancing HRQoL measures in the elderly of two communities in Brazil.

## Data Availability Statement

The raw data supporting the conclusions of this article will be made available by the authors, without undue reservation.

## Ethics Statement

The studies involving human participants were reviewed and approved by The Institutional Ethical Committee of Anhembi Morumbi University and Brasil University approved this study (CAAE: 11818919.8.0000.5492 and 24558913.7.0000.5494, respectively). The patients/participants provided their written informed consent to participate in this study.

## Author Contributions

MP, SC, LC, and OB: study conception and design. MP and SC: performed the study. LC, OB, RM, GR, MP, and SC: assays and data analysis. OB and LC: interpretation of the data and writing of the manuscript. RM, GR, MP, and SC: Critical revision of the manuscript regarding the important intellectual content. All authors contributed to the article and approved the submitted version.

## Conflict of Interest

The authors declare that the research was conducted in the absence of any commercial or financial relationships that could be construed as a potential conflict of interest.
